# Chronic Microcystin-LR Exposure Induces Colorectal Inflammation Associated with Macrophage Infiltration and HIF-1α-Related Glycolytic Reprogramming

**DOI:** 10.3390/toxics14100899

**Published:** 2026-10-10

**Authors:** Yan Tang, Xiaodie Zhou, Langyi Li, Chunhua Zhan, Fei Yang

**Affiliations:** 1 Key Laboratory of En-vironmental Health and Risk Control of Hunan Province, Hengyang Medical School, University of South China, Hengyang 421009, China; 2 Affiliated Nanhua Hospital, Hengyang Medical School, University of South China, Hengyang 421009, China

**Keywords:** microcystin-LR, chronic exposure, colorectal inflammation, macrophage infiltration, HIF-1α, glycolytic reprogramming

## Abstract

Microcystin-LR (MC-LR), one of the most prevalent cyanobacterial toxins in freshwater environments, has raised increasing concern because of its potential health risks asso-ciated with long-term exposure through contaminated drinking water and food, as well as inhalation of bloom-derived aerosols. Although intestinal toxicity has emerged as an important consequence of MC-LR exposure, the mechanisms underlying chronic colorectal inflammation remain incompletely understood. In the present study, mice were continuously exposed to MC-LR at 0, 1, 60, or 120 μg/L through drinking water for 15 months to investigate the effects of prolonged exposure on colorectal injury, inflammatory responses, macrophage infiltration, and glycolytic metabolism. Chronic MC-LR exposure did not significantly alter body weight or water consumption, whereas exposure to 60 and 120 μg/L MC-LR significantly shortened colon length. Histopathological examination revealed pronounced colorectal mucosal injury in the 60 and 120 μg/L groups, characterized by mucosal epithelial cell shedding, inflammatory cell infiltration, and disorganization of the crypt architecture. Consistently, MC-LR exposure markedly increased the expression of the pro-inflammatory cytokines *IL-1β*, *IL-6*, and *TNF-α* and promoted the accumulation of F4/80-positive macrophages in colorectal tissues. Given that inflammatory macrophage activation is closely coupled to glycolysis-associated metabolic remodeling and that HIF-1α serves as a key transcriptional regulator linking inflammatory responses to glycolytic metabolism, we further examined HIF-1α and related metabolic markers. Prolonged MC-LR exposure increased HIF-1α expression together with the glycolysis-associated proteins HK2, PKM, and LDHA and was accompanied by increased lactate accumulation, indicating a glycolysis-associated metabolic phenotype in colorectal tissues. Collectively, these findings suggest that chronic MC-LR exposure may promote colorectal toxicity through a potential sequential inflammatory–HIF-1α–glycolytic remodeling axis, in which macrophage-associated inflammation is accompanied by HIF-1α upregulation and subsequent glycolysis-associated metabolic reprogramming. This immunometabolic alteration may further contribute to the persistence of colorectal inflammation and tissue injury during long-term MC-LR exposure.

## 1. Introduction

Harmful cyanobacterial blooms have become an increasingly important environmental and public health concern worldwide, primarily driven by eutrophication and climate change [1,2]. Microcystins (MCs), a family of cyclic heptapeptide cyanotoxins produced by several bloom-forming cyanobacterial species, are among the most frequently detected and toxic cyanobacterial metabolites. Among the numerous MC congeners, microcystin-LR (MC-LR) has received particular attention because of its widespread occurrence, high toxicity, and potential for human exposure through contaminated drinking water and food [3]. MC-LR is primarily produced by toxin-producing cyanobacteria during harmful cyanobacterial blooms. Nutrient enrichment, particularly increased nitrogen and phosphorus loading, together with favorable environmental conditions such as elevated temperature and water-column stability, can promote cyanobacterial proliferation and increase the occurrence of MC-LR in lakes, reservoirs, rivers, and other surface waters. Human exposure to MC-LR may subsequently occur through consumption of contaminated drinking water, ingestion of aquatic organisms such as fish and shellfish in which MC-LR may accumulate, and consumption of edible crops irrigated with MC-LR-contaminated water [4]. Although the liver is generally regarded as the primary target organ of MC-LR, accumulating evidence indicates that the gastrointestinal tract is also highly susceptible to MC-LR toxicity because it represents a major interface for orally ingested contaminants [5,6,7,8]. Experimental studies have demonstrated that prolonged MC-LR exposure disrupts intestinal architecture, alters inflammatory mediator expression, and compromises epithelial barrier integrity [6,7]. Importantly, long-term exposure to environmentally relevant levels of MC-LR has been reported to induce chronic colorectal inflammation, fibrosis, inflammatory cell infiltration, and barrier disruption in mice, highlighting the colorectum as an important target of chronic MC-LR toxicity [8]. Nevertheless, the cellular and metabolic mechanisms that sustain colorectal inflammation during prolonged MC-LR exposure remain incompletely understood [9].

Macrophages are central regulators of intestinal immune homeostasis and represent an important cellular component of chronic inflammatory responses. In response to environmental stressors and tissue injury, recruited and resident macrophages can undergo marked phenotypic and functional remodeling and produce inflammatory mediators such as interleukin (IL)-1β, IL-6, and tumor necrosis factor (TNF)-α. Increasing evidence suggests that macrophage function is tightly coupled to cellular metabolism. In particular, inflammatory macrophage activation is frequently accompanied by a metabolic shift toward enhanced glycolytic metabolism, a process commonly referred to as immunometabolic reprogramming [10,11]. Hypoxia-inducible factor-1α (HIF-1α) is a pivotal transcriptional regulator linking metabolic adaptation to inflammatory activation. Stabilization of HIF-1α promotes the expression of glycolysis-related genes and facilitates inflammatory cytokine production, whereas metabolic intermediates generated during macrophage activation can further reinforce HIF-1α-dependent inflammatory signaling. For example, succinate accumulation in activated macrophages stabilizes HIF-1α and promotes IL-1β production [12]. Moreover, pyruvate kinase M2 (PKM2), a key glycolytic enzyme, can interact with HIF-1α and enhance the transcription of HIF-1α-responsive genes, thereby coupling glycolytic reprogramming to IL-1β production and the pro-inflammatory macrophage phenotype [13]. Other glycolysis-associated regulators, including PFKFB3, PDK1, hexokinase, and LDHA, also participate in the metabolic adaptation of inflammatory macrophages [10]. These findings establish the HIF-1α–glycolysis axis as an important mechanistic link between cellular metabolism and persistent inflammation.

Despite growing evidence for MC-LR-induced intestinal inflammation, whether metabolic reprogramming is involved in the chronic colorectal inflammatory response to MC-LR remains largely unclear. Previous studies have predominantly focused on oxidative stress, epithelial barrier dysfunction, gut microbiota dysbiosis, inflammasome activation, and inflammatory signaling pathways in MC-LR-induced intestinal injury [14], whereas the potential involvement of macrophage-associated glycolytic alterations has received considerably less attention. Therefore, the present study established a long-term MC-LR exposure model in mice by administering 0, 1, 60, or 120 μg/L MC-LR in drinking water for 15 months. We investigated colorectal morphological and histopathological alterations, the expression of major pro-inflammatory cytokines, macrophage infiltration, and changes in HIF-1α and key glycolysis-associated molecules. We hypothesized that prolonged MC-LR exposure induces chronic colorectal injury accompanied by macrophage accumulation and HIF-1α-associated glycolytic reprogramming. In contrast to our previous study, which focused primarily on colorectal barrier disruption, fibrosis, and CSF1R/Rap1b signaling, the present study was designed to investigate the immunometabolic alterations associated with more prolonged MC-LR exposure, with particular emphasis on HIF-1α-related glycolytic reprogramming [8]. This study aimed to provide new insight into the immunometabolic mechanisms underlying chronic intestinal toxicity following long-term environmental exposure to MC-LR.

## 2. Materials and Methods

### 2.1. Chemicals, Animals, and Experimental Design

Microcystin-LR (MC-LR; purity ≥ 95%) was purchased from Alexis Corporation (Lausen, Switzerland). MC-LR was dissolved in ultrapure water to prepare a stock solution at a nominal concentration of 1 mg/mL. The stock solution was aliquoted, protected from light, and stored at −80 °C until use. Before administration, the stock solution was diluted with drinking water to the designated exposure concentrations. MC-LR-containing drinking water was freshly prepared and replaced twice weekly throughout the 15-month exposure period. To minimize potential photodegradation, the drinking water was maintained in brown bottles wrapped with aluminum foil and kept under light-protected conditions. The MC-LR concentrations in the drinking water were periodically measured using an ELISA (Beacon, NY, USA) kit to confirm that the actual concentrations remained consistent with the intended exposure levels throughout the study.

Fifty-two specific-pathogen-free (SPF) male C57BL/6J mice, aged 6–8 weeks and weighing 20–22 g, were obtained from Hunan SJA Laboratory Animal Co., Ltd. (Changsha, China). The exposure concentrations were selected with reference to the toxicological assessment of MC-LR reported by Fawell et al., in which a no-observed-adverse-effect level (NOAEL) of 40 μg/kg body weight/day was identified following oral administration. Based on the corresponding dose-conversion approach, an equivalent drinking-water exposure concentration of approximately 250 μg/L was estimated [15,16]. Accordingly, 120 and 60 μg/L were selected to represent approximately one-half and one-quarter of this reference concentration, respectively. In addition, a concentration of 1 μg/L was included because this value corresponds to the guideline/standard limit for MC-LR in drinking water established by the World Health Organization and the Chinese Standards for Drinking Water Quality, thereby allowing evaluation of the potential biological effects associated with long-term exposure at the current regulatory threshold [3]. Because the mice were group-housed, individual water consumption could not be directly measured. Water intake was therefore recorded on a cage basis. The total water consumption of each cage was determined from the difference in drinking-bottle weight before and after the measurement period, and the average water intake per mouse was estimated by dividing the total cage consumption by the number of mice housed in that cage. Thus, the reported water-consumption values represent cage-based estimates rather than direct measurements from individual animals.

The mice were randomly assigned to four groups using a random-number method, with 13 animals per group: a control group receiving MC-LR-free drinking water, and three MC-LR exposure groups receiving drinking water containing 1 μg/L, 60 μg/L, or 120 μg/L MC-LR. A total of 13 animals per group were included in the chronic exposure cohort. Because colorectal tissues were allocated to different downstream analyses, assay-specific sample sizes varied among experiments. Unless otherwise stated, the reported n values represent independent biological replicates rather than technical replicates. No animals or data points were excluded based on experimental outcomes. All animals were exposed continuously for 15 months through ad libitum consumption of the corresponding drinking water. Water intake was recorded periodically throughout the exposure period to characterize MC-LR consumption, and body weight was monitored regularly during the experiment. The animals used in the present study constituted an independent experimental cohort established within our long-term MC-LR exposure model platform and were distinct from those used in our previously published study [8]. No biological samples from the previous study were reused in the present investigation.

At the end of the 15-month exposure period, the mice were fasted for 12 h and weighed. Animals were then euthanized by overdose inhalation of isoflurane. Approximately the proximal 1 cm segment of the colon, immediately distal to the cecum, was collected for subsequent histological and molecular analyses. All animal procedures were conducted in accordance with the ethical requirements for laboratory animal research of the Xiangya School of Public Health, Central South University. The experimental protocol was reviewed and approved by the Ethics Committee of the Xiangya School of Public Health, Central South University in June 2018 (Permit No. XYGW-2018-41). The animal exposure experiment commenced in July 2018, and the entire 15-month experimental period was conducted within the scope and validity of the approved protocol.

### 2.2. Histopathological Examination and Immunohistochemistry

Colorectal tissues were carefully isolated and spread flat on filter paper for gross morphological examination. The tissues were rinsed three times with pre-cooled physiological saline, and residual surface liquid was gently removed using filter paper. A colorectal segment was collected approximately 1 cm proximal to the anal verge and gently rolled longitudinally around a thin rod to form a Swiss-roll configuration. The rolled tissues were then fixed in 4% paraformaldehyde at room temperature for 24 h.

For histopathological examination, fixed colorectal tissues were dehydrated through a graded ethanol series, cleared in xylene, embedded in paraffin, and sectioned. The sections were subsequently deparaffinized, rehydrated, and stained with hematoxylin and eosin (H&E). Histopathological alterations in the colorectal mucosa, including epithelial integrity, crypt architecture, and mucosal structure, were examined under a light microscope.

For immunohistochemical analysis, paraffin-embedded colorectal sections were deparaffinized and rehydrated, followed by antigen retrieval and blocking of endogenous peroxidase activity. After blocking nonspecific binding, the sections were incubated with an anti-F4/80 primary antibody to identify macrophages, followed by incubation with an appropriate horseradish peroxidase-conjugated secondary antibody. Immunoreactivity was visualized using 3,3′-diaminobenzidine (DAB), and the nuclei were counterstained with hematoxylin. F4/80-positive cells were examined under a light microscope and used to evaluate macrophage infiltration in colorectal tissues. For quantitative analysis of F4/80 immunohistochemistry, six randomly selected, non-overlapping microscopic fields were evaluated for each tissue sample at 40× magnification. F4/80 immunoreactivity was quantified using ImageJ software (v1.46r, National Institutes of Health, Bethesda, MD, USA) as the percentage of F4/80-positive area within each microscopic field. The mean percentage of positive area from the six fields was calculated for each biological sample. Identical image-acquisition conditions and quantification criteria were applied to all experimental groups.

### 2.3. Quantitative Real-Time Pcr Analysis

Total RNA was extracted from colorectal tissues using a commercial RNA extraction reagent according to the manufacturer’s instructions. RNA concentration and purity were determined spectrophotometrically, and RNA integrity was further assessed by agarose gel electrophoresis. Only RNA samples with A260/A280 ratios between 1.8 and 2.1 and clearly resolved 28S and 18S rRNA bands were used for subsequent analyses. Equal amounts of total RNA were reverse-transcribed into cDNA using HiScript II Q RT SuperMix for qPCR (+gDNA wiper) (Cat. No. R223-01, Vazyme Biotech Co., Ltd., Nanjing, China) according to the manufacturer’s instructions. Quantitative real-time PCR was performed using ChamQ Universal SYBR qPCR Master Mix (Cat. No. Q711-02, Vazyme Biotech Co., Ltd., Nanjing, China). Primer amplification efficiencies were validated by standard-curve analysis before use, and only primer pairs showing acceptable amplification performance and specificity were used for gene-expression analysis. Each biological sample was analyzed in three technical replicates. *β-actin* was used as the reference gene, and its stability was confirmed by comparing Ct values across all experimental groups; no significant differences in *β-actin* Ct values were observed among the control and MC-LR exposure groups. Relative mRNA expression levels were calculated using the 2^−ΔΔCt^ method. The primer sequences used for qPCR are listed in Table 1. Six independent biological samples were analyzed per group (*n* = 6). Each biological sample was measured in three technical replicates, which were averaged to yield a single value for statistical analysis.

### 2.4. Western Blot Analysis

Total protein was extracted from colorectal tissues using ice-cold RIPA lysis buffer supplemented with protease and phosphatase inhibitors. Protein concentrations were determined using a BCA protein assay kit. Equal amounts of total protein (40 μg per lane) were separated by SDS-PAGE using a 5% stacking gel and 10–12% resolving gels, with the resolving-gel percentage selected according to the molecular weight of the target protein. Electrophoresis was performed at 80 V through the stacking gel and subsequently at 120 V through the resolving gel. Proteins were transferred onto PVDF membranes using a wet-transfer system at a constant voltage of 100 V for up to 120 min, with the transfer duration adjusted according to the molecular weight of the target protein. The membranes were blocked with 5% non-fat milk in TBST for 2 h at room temperature and subsequently incubated overnight at 4 °C with the following primary antibodies: β-actin (Cell Signaling Technology, Danvers, MA, USA, Cat. No. 4967, 1:1000), HK2 (Proteintech, Wuhan, Hubei, China, Cat. No. 22029-1-AP, 1:5000), PKM (Proteintech, Cat. No. 25659-1-AP, 1:2000), LDHA (Proteintech, Cat. No. 21799-1-AP, 1:10,000), HIF-1α (Abiowell, Changsha, Hunan, China, Cat. No. 47C02, 1:3000), and Total OXPHOS Rodent WB Antibody Cocktail (Abcam, Cambridge, UK, Cat. No. ab110413, 1:1000). The MTCO1 band contained in the Total OXPHOS antibody cocktail was used to assess mitochondrial respiratory-chain complex IV. After washing with TBST, the membranes were incubated with the corresponding HRP-conjugated secondary antibodies for 1 h at room temperature. Protein signals were developed using an ultrasensitive enhanced chemiluminescence reagent (Abbkine, BMU102-CN) and captured using a MiniChemi610Plus chemiluminescence imaging system (Beijing, China) under non-saturating exposure conditions. Band intensities were quantified using ImageJ software and normalized to β-actin.

### 2.5. Statistical Analysis

Data processing and statistical analyses were performed using Microsoft Excel and SPSS Statistics version 26.0 (IBM Corp., Armonk, NY, USA). The normality of continuous variables was assessed using the Shapiro–Wilk test, and homogeneity of variances was evaluated using Levene’s test. Data satisfying the assumptions of normality and homogeneity of variance were analyzed using one-way analysis of variance (ANOVA), followed by Dunnett’s post hoc test to compare each MC-LR exposure group with the control group. For body-weight data, the statistical objective was to compare the experimental groups at each individual measurement time point rather than to assess longitudinal changes or a treatment × time interaction. Therefore, body-weight data at each time point were analyzed separately using one-way ANOVA followed by Dunnett’s post hoc test, with each MC-LR exposure group compared with the concurrent control group. For data that did not meet the assumptions required for parametric analysis, the Kruskal–Wallis H test was applied, followed by post hoc comparisons between each exposure group and the control group when an overall significant difference was detected. All statistical tests were two-sided, and a *p*-value < 0.05 was considered statistically significant.

## 3. Results

### 3.1. Chronic MC-LR Exposure Induces Colorectal Injury in Mice

**Figure 1:**
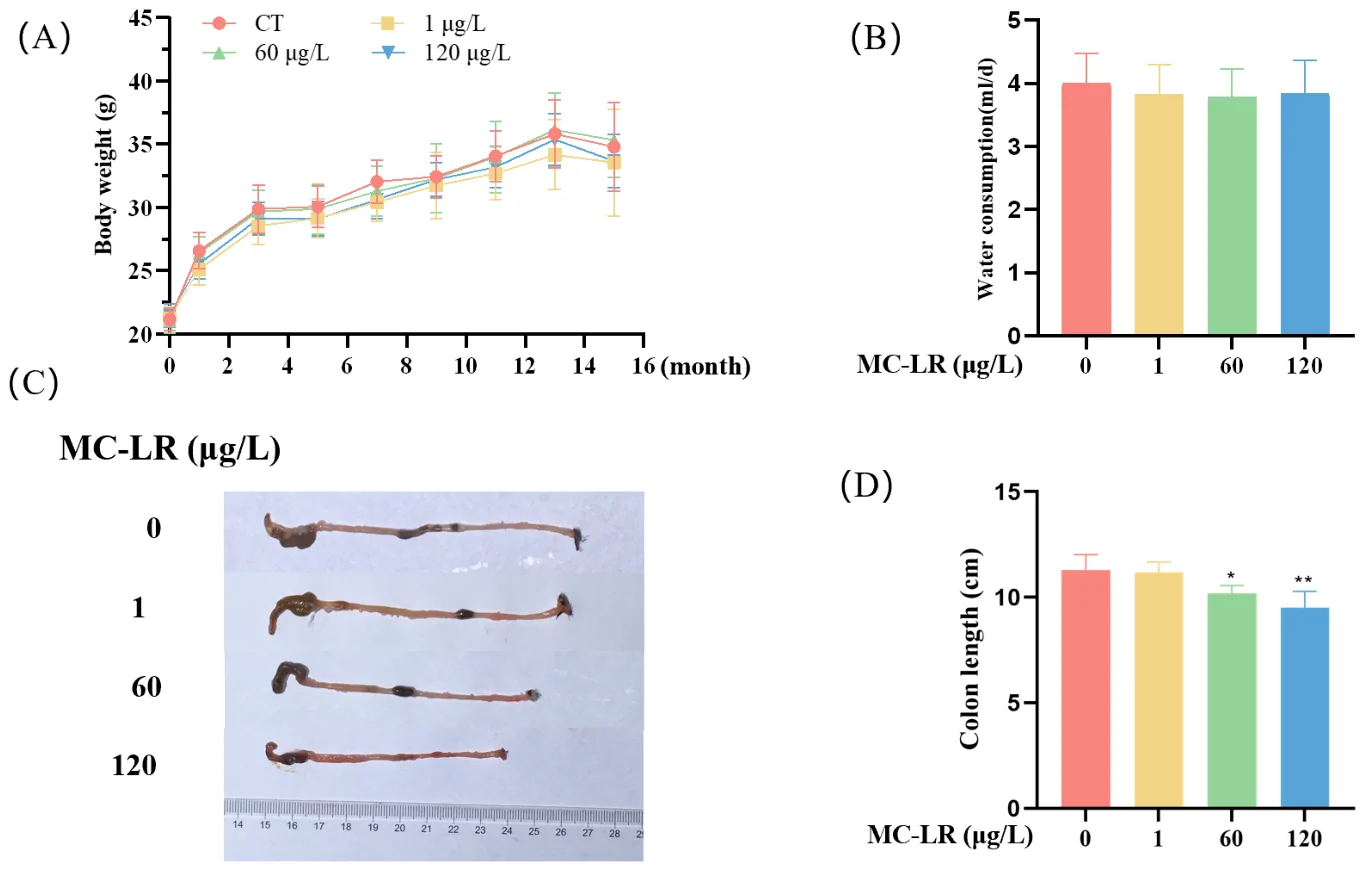
Effects of chronic MC-LR exposure on the colorectum of mice. (**A**) Dynamic changes in body weight during the 15-month exposure period. (**B**) Average daily water intake of mice in each group. (**C**) Representative images of the colorectum from mice in the control and MC-LR exposure groups. (**D**) Quantification of colorectal length. Data are presented as mean ± SEM (*n* = 13). Statistical differences were analyzed using one-way analysis of variance (ANOVA), followed by Dunnett’s post hoc test for comparisons between each MC-LR exposure group and the control group. * *p* < 0.05; ** *p* < 0.01.

During the 15-month exposure period, body weight was monitored regularly in all groups. Mice in all groups exhibited a progressive increase in body weight, with the most rapid gain occurring during the first two months, followed by a gradual slowing of weight gain and subsequent stabilization. Although body weight progressively increased during the early phase of the experiment and subsequently stabilized, comparisons performed separately at each measurement time point revealed no significant differences between the MC-LR-exposed groups and the concurrent control group (Figure 1A). MC-LR-containing drinking water was freshly prepared and replaced twice weekly throughout the 15-month exposure period. Daily water consumption remained relatively stable at approximately 3.9 mL per mouse, with no significant differences among the groups. Based on the recorded water intake, the estimated daily MC-LR intake was 0.0038, 0.229, and 0.46 μg/mouse/day in the 1, 60, and 120 μg/L exposure groups, respectively (Figure 1B). After 15 months of exposure, gross examination of the colorectum revealed a reduction in colon length in MC-LR-exposed mice. Compared with the control group, mice exposed to 60 and 120 μg/L MC-LR exhibited a significant shortening of the colon (Figure 1C,D), indicating that prolonged MC-LR exposure adversely affected colorectal morphology.

### 3.2. Long-Term MC-LR Exposure Induces Colorectal Injury in Mice

**Figure 2:**
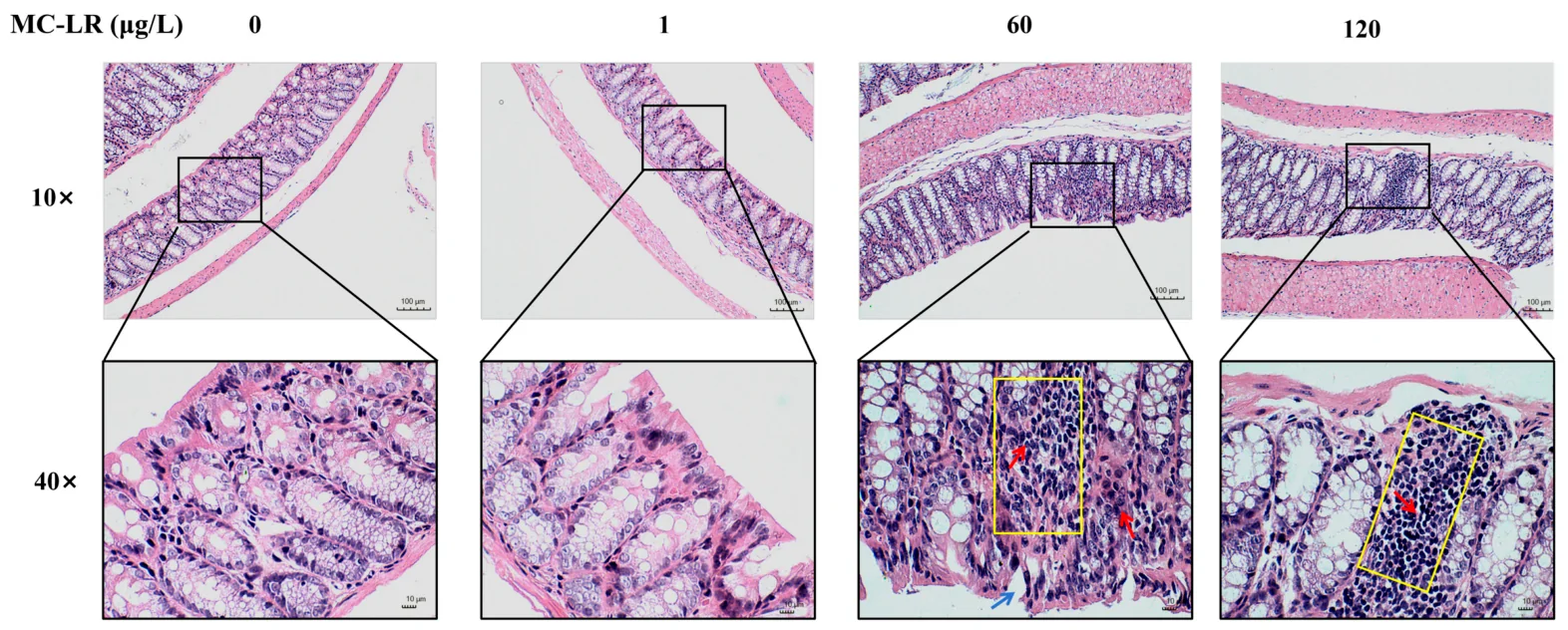
Histopathological alterations in the colorectal tissues of mice following chronic MC-LR exposure. Representative H&E-stained sections of colorectal tissues from the control and MC-LR-exposed groups. Chronic MC-LR exposure resulted in prominent histopathological alterations, including mucosal epithelial cell shedding, inflammatory cell infiltration, and disorganization of the crypt architecture. Red arrows indicate lymphocyte infiltration; yellow boxes highlight regions with abnormal arrangement of crypt glands; and blue arrows indicate disruption of mucosal epithelial continuity accompanied by superficial epithelial exfoliation. Images were acquired at 10× magnification (scale bar = 100 μm) and 40× magnification (scale bar = 10 μm).

Histopathological alterations in colorectal tissues were evaluated by H&E staining. The colorectal mucosa of control mice exhibited an intact tissue architecture, characterized by regularly arranged epithelial cells, well-preserved glandular and crypt structures, and a normal mucosal thickness, with no apparent pathological abnormalities. In contrast, mice exposed to 60 and 120 μg/L MC-LR displayed pronounced histological alterations. Chronic MC-LR exposure induced prominent histopathological alterations in the colorectal tissue, characterized by mucosal epithelial cell shedding, inflammatory cell infiltration, and disorganization of the crypt architecture. Collectively, these histopathological changes indicated that long-term MC-LR exposure caused substantial colorectal mucosal injury accompanied by features consistent with chronic intestinal inflammation (Figure 2).

### 3.3. Long-Term MC-LR Exposure Induces Chronic Colorectal Inflammation in Mice

**Figure 3:**
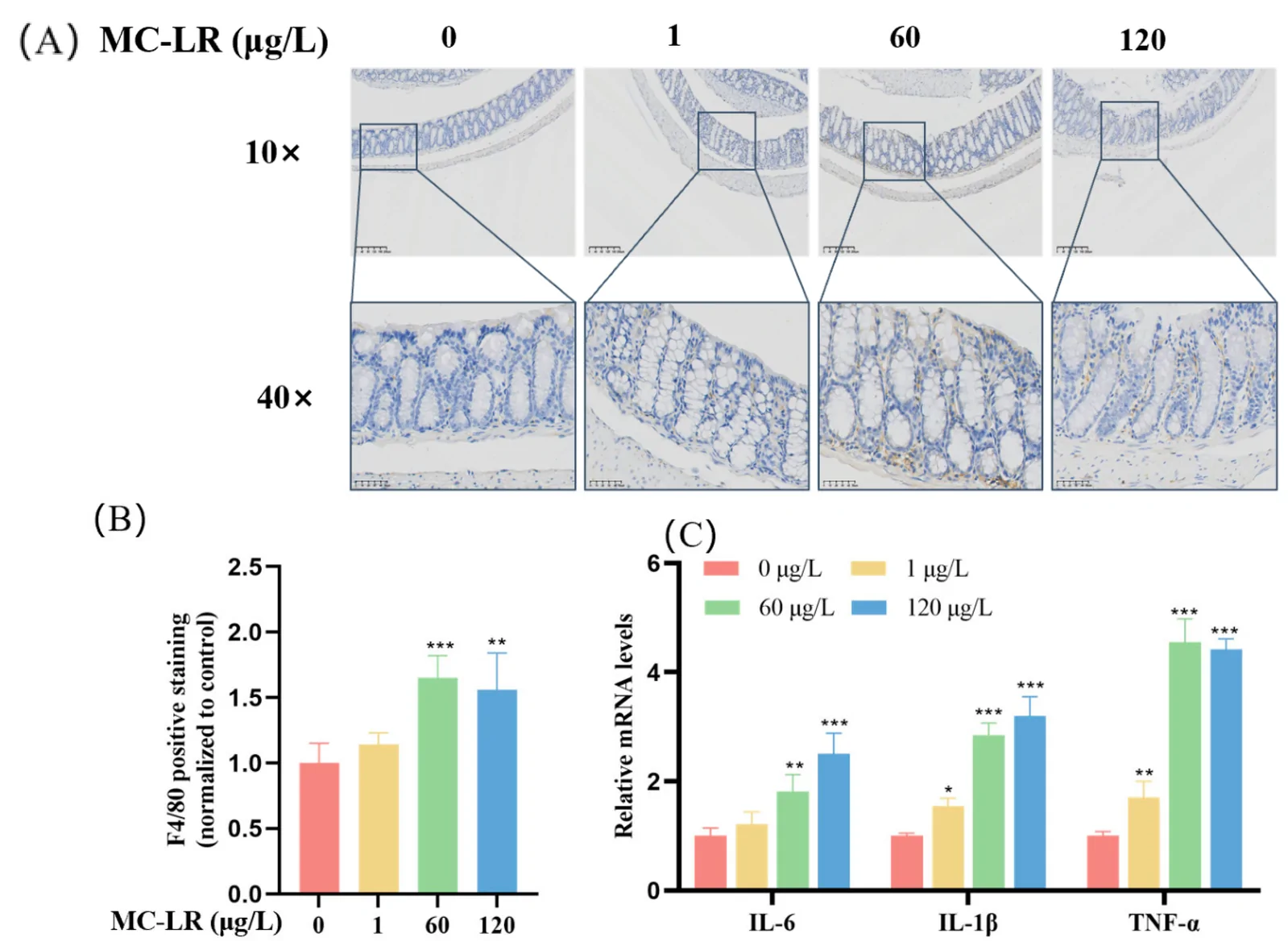
Chronic MC-LR exposure induces colorectal inflammation and macrophage infiltration in mice. (**A**,**B**) Representative immunohistochemical staining of F4/80-positive macrophages in colorectal tissues from the control and MC-LR-exposed groups. Chronic MC-LR exposure increased macrophage infiltration in the colorectal mucosa. Images were acquired at 10× magnification (scale bar = 200 μm) and 40× magnification (scale bar = 50 μm). (**C**) Relative mRNA expression levels of pro-inflammatory cytokines in colorectal tissues, including *IL-1β*, *IL-6*, and *TNF-α*. Data are presented as mean ± SEM (*n* = 6). Statistical differences were analyzed using one-way analysis of variance (ANOVA), followed by Dunnett’s post hoc test for comparisons between each MC-LR exposure group and the control group. * *p* < 0.05; ** *p* < 0.01; *** *p* < 0.001.

To further characterize the inflammatory response induced by long-term MC-LR exposure, the mRNA expression levels of the pro-inflammatory cytokines *IL-1β*, *IL-6*, and *TNF-α* were determined in colorectal tissues. Overall, MC-LR exposure resulted in a concentration-related elevation of these inflammatory mediators (Figure 3C). Compared with the control group, *IL-6* expression was significantly increased to 1.82- and 2.51-fold in mice exposed to 60 and 120 μg/L MC-LR, respectively (*p* < 0.05). *IL-1β* expression was significantly elevated at all three exposure concentrations, reaching 1.54, 2.85, and 3.20-fold of the control level in the 1, 60, and 120 μg/L groups, respectively (*p* < 0.05). Similarly, TNF-α expression was markedly increased by 1.71, 4.56, and 4.42-fold in the 1, 60, and 120 μg/L MC-LR groups, respectively, compared with the control group (*p* < 0.05).

Immunohistochemical staining was further performed to evaluate macrophage infiltration in colorectal tissues. As shown in Figure 3A,B, the number of F4/80-positive macrophages was significantly increased following MC-LR exposure, with abundant F4/80-positive cells distributed throughout the colorectal mucosal and epithelial regions. These findings, together with the elevated expression of pro-inflammatory cytokines, demonstrate that long-term MC-LR exposure induces a persistent inflammatory response in the mouse colorectum characterized by enhanced macrophage infiltration and increased production of inflammatory mediators.

### 3.4. Chronic MC-LR Exposure Disrupts Energy Metabolic Homeostasis in Mouse Colorectal Tissues

**Figure 4:**
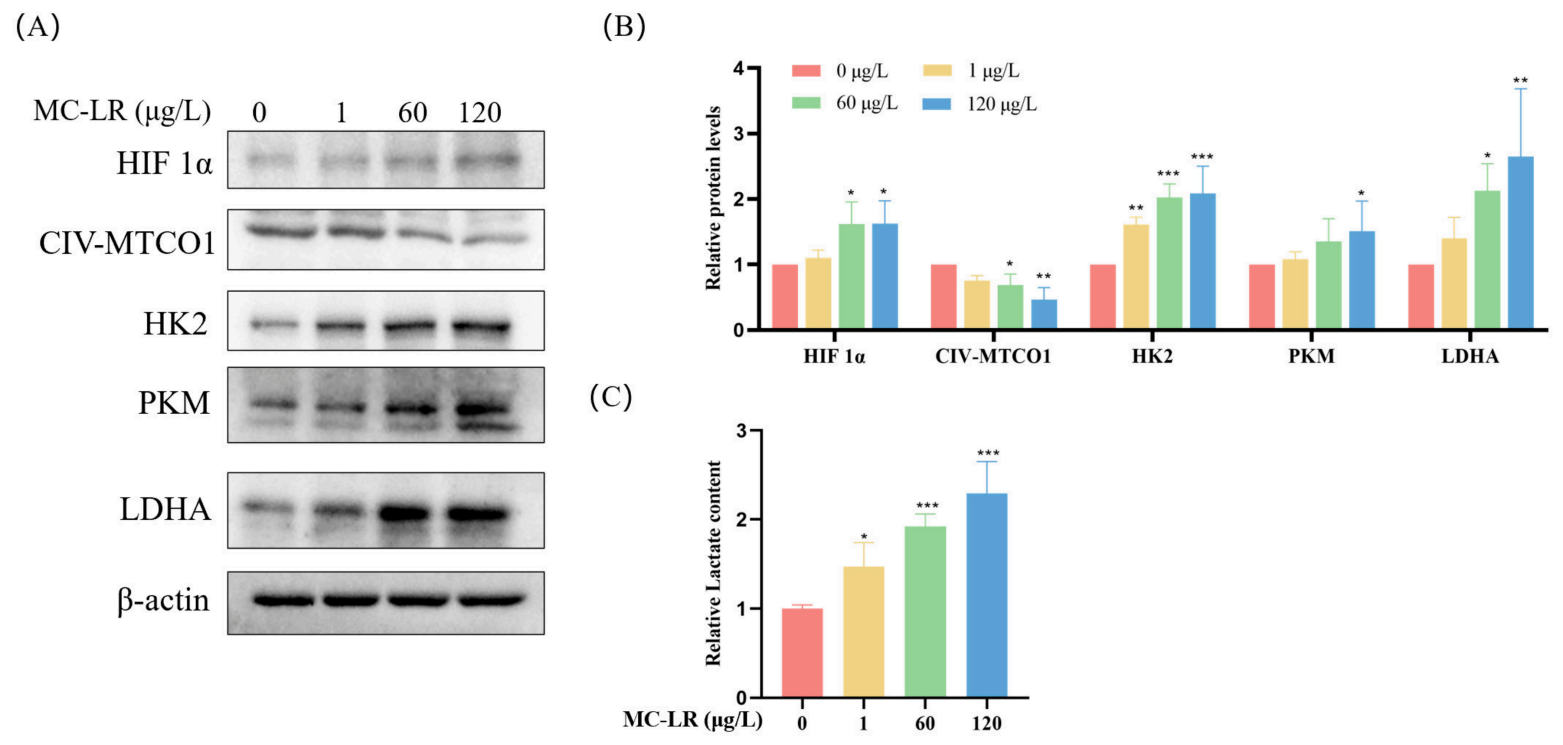
Chronic MC-LR exposure alters energy metabolism in mouse colorectal tissues. (**A**,**B**) Western blot analysis and quantitative assessment of HIF-1α, HK2, PKM, LDHA, and CIV-MTCO1 protein expression in colorectal tissues from the control and MC-LR-exposed groups. β-actin was used as the loading control. (**C**) Lactate levels in colorectal tissues following chronic MC-LR exposure. Data are presented as mean ± SEM (*n* = 3). Statistical differences were analyzed using one-way analysis of variance (ANOVA), followed by Dunnett’s post hoc test to compare each MC-LR exposure group with the control group. * *p* < 0.05; ** *p* < 0.01; *** *p* < 0.001.

Western blot analysis was performed to evaluate the effects of chronic MC-LR exposure on energy metabolism in colorectal tissues, with particular emphasis on key glycolysis-associated proteins, including hexokinase 2 (HK2), pyruvate kinase (PKM), and lactate dehydrogenase A (LDHA), as well as the mitochondrial respiratory chain complex IV subunit MTCO1 (CIV-MTCO1). As shown in Figure 4, long-term MC-LR exposure induced marked metabolic alterations in colorectal tissues. Compared with the control group, the protein expression levels of HK2, PKM, and LDHA were significantly increased in the MC-LR-exposed groups, whereas CIV-MTCO1 expression showed a concentration-dependent decrease. In addition, HIF-1α protein levels were significantly elevated in the 60 and 120 μg/L MC-LR groups.

To further evaluate changes in glycolytic metabolism, lactate levels were measured in colorectal tissues. The results showed that lactate accumulation was increased in the MC-LR-exposed groups compared with the control group (Figure 4B), consistent with the upregulation of glycolysis-associated proteins. Collectively, these findings indicate that chronic MC-LR exposure is associated with a glycolysis-associated metabolic phenotype, characterized by increased HIF-1α, HK2, PKM, LDHA, and lactate together with reduced MTCO1 expression. These molecular and biochemical alterations suggest disturbed colorectal energy metabolic homeostasis but do not directly demonstrate increased glycolytic flux.

## 4. Discussion

The present study demonstrates that prolonged exposure to MC-LR through drinking water produces persistent colorectal injury and inflammatory alterations in mice and, importantly, associates these pathological effects with substantial remodeling of cellular energy metabolism. Following 15 months of exposure, MC-LR did not significantly affect overall body-weight trajectories or water consumption, suggesting that the intestinal alterations observed were unlikely to be secondary to overt systemic toxicity or differences in fluid intake [17]. Nevertheless, mice exposed to the highest concentration of MC-LR exhibited significant colon shortening, together with marked histopathological alterations in the colorectum, including mucosal epithelial cell shedding, inflammatory cell infiltration, and disorganization of the crypt architecture. These findings are consistent with previous evidence identifying the gastrointestinal tract as an important target of chronic MC-LR toxicity. Cao et al. reported that six-month exposure to MC-LR caused marked structural injury and altered inflammatory mediator expression in the mouse jejunum [6]. Similarly, long-term exposure to MC-LR at a regulatory-level concentration and higher experimental concentrations has been shown to induce colorectal epithelial barrier disruption, inflammatory cell infiltration, fibrosis, and chronic inflammation in mice [8]. Notably, the same study also demonstrated marked alterations in the gut microbiota and microbiota-related metabolites, suggesting that disruption of the microbiota–barrier axis may participate in chronic MC-LR-induced colorectal injury. Consistent with this concept, subsequent studies have further linked MC-LR exposure to reduced intestinal tight-junction protein expression and gut microbiota dysbiosis. Although the present study revealed clear histopathological evidence of mucosal epithelial injury, epithelial barrier integrity and gut microbiota composition were not directly assessed. MC-LR can also exacerbate pre-existing experimental colitis, as reflected by increased colon shortening, mucosal ulceration, and inflammatory cytokine production [7], while more recent evidence has implicated NLRP3 inflammasome-mediated pyroptosis in MC-LR-induced and MC-LR-aggravated colitis [14]. Collectively, these studies support the notion that chronic intestinal injury represents an important component of MC-LR toxicity. Our findings extend this evidence by showing that a very long exposure period can sustain colorectal injury even in the absence of marked alterations in body weight, indicating that local intestinal responses may precede or occur independently of overt systemic manifestations.

A prominent feature of the present study was the enhanced inflammatory response in colorectal tissues. MC-LR exposure markedly elevated the transcription of IL-1β, IL-6, and TNF-α, and these changes were accompanied by increased accumulation of F4/80-positive macrophages in the colorectal mucosa. Macrophage recruitment is increasingly recognized as an important component of MC-LR-associated intestinal pathology. Koto et al. demonstrated that environmentally relevant MC-LR exposure increased MCP-1 expression in rat colonic mucosa and that MCP-1 expression correlated positively with ADGRE1/F4/80, supporting a mechanism in which epithelial exposure to MC-LR promotes chemokine-dependent macrophage accumulation. Their in vitro experiments further showed that MC-LR entered colonic epithelial-like cells through probenecid-sensitive transport processes and activated the JNK/MCP-1 pathway [18]. In addition, microcystin exposure has been associated with increased intestinal F4/80 immunoreactivity, redox disturbances, microbiome alterations, and inflammasome activation in susceptible mice [19]. Macrophages are particularly relevant to chronic intestinal inflammation because they are not merely passive inflammatory infiltrates; their activation state determines cytokine production, epithelial injury, resolution, and tissue remodeling. Thus, the parallel increase in F4/80-positive macrophages and IL-1β, IL-6, and TNF-α observed here raises the possibility that macrophage accumulation contributes to maintenance of the inflammatory microenvironment during prolonged MC-LR exposure. However, because cytokine expression was measured in whole colorectal tissue, the present data do not establish macrophages as the exclusive cellular source of these mediators. Intestinal epithelial cells and other immune-cell populations may also contribute, and this distinction should be addressed in future studies using cell-specific isolation, flow cytometry, or co-localization approaches.

One of the most notable observations in this study was the coordinated alteration of glycolytic and mitochondrial markers. Chronic MC-LR exposure increased HIF-1α together with HK2, PKM, and LDHA, while colorectal lactate levels were elevated. In contrast, CIV-MTCO1, a core mitochondrially encoded subunit of respiratory-chain complex IV, progressively declined with increasing MC-LR exposure. This pattern is compatible with a metabolic shift characterized by greater reliance on glycolytic metabolism and reduced mitochondrial respiratory capacity. Such metabolic reprogramming is now recognized as an integral component of inflammatory immune-cell activation rather than merely a consequence of cellular stress [11]. Pro-inflammatory macrophages typically increase glucose uptake and glycolytic flux while reducing their dependence on mitochondrial oxidative phosphorylation, thereby rapidly generating ATP and metabolic intermediates required for inflammatory effector functions [10]. Integrated metabolic and transcriptional analyses have further shown that macrophage activation entails coordinated remodeling of glycolysis and the tricarboxylic acid cycle rather than a simple increase in energy demand [20]. Mitochondria themselves are also repurposed during inflammatory activation: succinate oxidation and altered electron transport can enhance mitochondrial ROS generation and support inflammatory gene expression [21]. In this context, the concomitant increase in glycolysis-associated proteins and lactate, together with reduced CIV-MTCO1 observed in our study, suggests that chronic MC-LR exposure may disrupt the balance between glycolytic and mitochondrial energy production in colorectal tissue. Importantly, this interpretation should be regarded as evidence of a metabolic phenotype rather than direct proof of glycolytic flux, because extracellular acidification rate, oxygen-consumption rate, isotope tracing, and mitochondrial respiratory function were not directly measured.

Although elevated lactate is consistent with altered glycolytic metabolism, lactate accumulation should not be interpreted in isolation because it may also reflect tissue hypoxia, impaired mitochondrial oxidation, altered lactate clearance, or cellular injury. In the present study, the increase in lactate was therefore interpreted together with the concurrent upregulation of HIF-1α, HK2, PKM, and LDHA and the reduction in CIV-MTCO1 expression. This combined metabolic profile is more consistent with disrupted energy homeostasis and a shift toward glycolysis-associated metabolism than with lactate accumulation alone. HIF-1α may represent an important molecular bridge between the metabolic and inflammatory changes observed in the present study. Beyond its canonical role in adaptation to hypoxia, HIF-1α is a central regulator of inflammatory myeloid-cell metabolism. Genetic studies demonstrated that HIF-1α is required for the glycolytic capacity, tissue infiltration, motility, and inflammatory function of myeloid cells [22]. HIF-1α can increase the expression of multiple glycolytic enzymes and redirect pyruvate metabolism away from mitochondrial oxidation, thereby supporting glycolysis under inflammatory conditions [23]. A particularly important mechanistic link between metabolism and inflammation was provided by Tannahill et al., who showed that succinate accumulation stabilizes HIF-1α and promotes IL-1β transcription in activated macrophages [12]. Moreover, PKM2 has functions extending beyond its catalytic role in glycolysis: the dimeric/monomeric form of PKM2 can interact with HIF-1α and promote HIF-1α-dependent transcription, including that of IL-1β and LDHA. Pharmacological activation of PKM2 toward its tetrameric configuration suppresses HIF-1α activity, glycolytic reprogramming, and IL-1β production in activated macrophages [13]. These established mechanisms provide a biologically plausible framework for our observation that HIF-1α elevation coincided with increased PKM, LDHA, lactate accumulation, and pro-inflammatory cytokine expression. Nevertheless, our data currently demonstrate association rather than direct pathway dependence. HIF-1α inhibition or genetic manipulation would be required to determine whether the observed glycolytic phenotype and inflammatory response are mechanistically dependent on HIF-1α during MC-LR exposure. Importantly, the role of HIF-1α in intestinal inflammation is highly context- and cell-type-dependent. In intestinal epithelial cells, HIF-1α has been shown to exert barrier-protective effects during experimental colitis, and epithelial HIF-1α deficiency aggravates disease severity [24,25,26]. In contrast, HIF-1α in myeloid cells supports glycolytic metabolism, inflammatory-cell function, and proinflammatory cytokine production, and myeloid HIF-1α deficiency can attenuate inflammatory responses in systemic inflammatory models [22]. Thus, HIF-1α should not be regarded as uniformly proinflammatory or anti-inflammatory. In the present study, HIF-1α was measured in whole colorectal tissue; therefore, the relative contributions of epithelial and immune-cell HIF-1α cannot be distinguished. Accordingly, our findings are interpreted as an association between HIF-1α-related metabolic remodeling and chronic colorectal inflammation rather than evidence of a direct proinflammatory role of HIF-1α.

The increase in lactate observed in the colorectal tissue provides an additional layer linking altered metabolism to persistent inflammation. Lactate was historically regarded primarily as an end product of glycolysis; however, it is now recognized as a metabolically active signaling molecule capable of modifying immune-cell behavior. Increased lactate production is expected when pyruvate is preferentially converted to lactate by LDHA rather than entering mitochondrial oxidative metabolism. In activated innate immune cells, enhanced glycolysis provides both rapid ATP production and intermediates required for biosynthetic and inflammatory processes [27,28]. Moreover, lactate can regulate transcriptional programs through mechanisms including histone lysine lactylation [29]. Zhang et al. identified histone lactylation as a metabolically derived epigenetic modification in macrophages and demonstrated that intracellular lactate accumulation can influence gene-expression programs during inflammatory activation [30]. The biological effects of lactate are nevertheless context-dependent; depending on concentration, exposure duration, cell type, and tissue microenvironment, lactate may participate in either inflammatory amplification or subsequent resolution and reparative responses. Accordingly, the increased colorectal lactate found here should not be interpreted as an inflammatory mediator by itself. Instead, together with increased HK2, PKM, and LDHA, it provides functional support for enhanced glycolytic metabolism. Determining whether lactate directly participates in MC-LR-induced intestinal inflammation will require analysis of lactate transporters, lactate receptor signaling, and potentially histone lactylation in specific intestinal cell populations.

Our results also suggest that mitochondrial dysfunction and enhanced glycolysis may be mechanistically interconnected rather than independent effects of MC-LR. In inflammatory macrophages, suppression or remodeling of oxidative phosphorylation can reinforce dependence on glycolysis and contribute to maintenance of inflammatory phenotypes. Van den Bossche et al. demonstrated that impaired mitochondrial oxidative phosphorylation constrains the ability of inflammatory macrophages to repolarize toward an anti-inflammatory state, suggesting that mitochondrial dysfunction can stabilize inflammatory cellular programs [31]. Conversely, HIF-1α can enhance glycolytic metabolism while limiting pyruvate entry into mitochondrial oxidation, thereby further consolidating a glycolytic phenotype. Our finding that CIV-MTCO1 decreased while HIF-1α, glycolytic proteins, and lactate increased is therefore compatible with a coordinated remodeling of energy metabolism. Previous studies have identified oxidative stress as an important component of MC-LR toxicity and have linked chronic exposure to redox imbalance, epithelial barrier disruption, inflammatory signaling, and mitochondrial injury [32]. However, oxidative stress markers were not directly assessed in the present study. Therefore, although redox disturbances may theoretically interact with HIF-1α signaling and metabolic remodeling, the involvement of oxidative stress in the metabolic phenotype observed here remains to be experimentally established. Future experiments assessing mitochondrial membrane potential, ATP production, ROS, oxygen-consumption rate, and electron-transport-chain activity will be important for defining whether mitochondrial impairment precedes HIF-1α activation and glycolytic adaptation during chronic MC-LR exposure.

## 5. Conclusions

Taken together, the present findings support a working model in which prolonged MC-LR exposure causes persistent colorectal epithelial injury and promotes recruitment of macrophages, accompanied by increased expression of inflammatory mediators. At the same time, colorectal energy metabolism shifts toward a more glycolytic phenotype, as indicated by increased HIF-1α, HK2, PKM, LDHA, and lactate, together with reduced CIV-MTCO1 expression. Considering the well-established role of HIF-1α-dependent glycolytic remodeling in inflammatory macrophages, it is reasonable to propose that immunometabolic reprogramming may contribute to the persistence of MC-LR-induced colorectal inflammation. This interpretation also expands the current mechanistic framework of MC-LR intestinal toxicity beyond epithelial injury, oxidative stress, inflammasome activation, and microbiota disturbance toward the integration of cellular metabolism and inflammatory regulation. Recent evidence showing that inhibition of the glycolytic regulator PFKFB3 in macrophages attenuates experimental colitis further supports the concept that macrophage glycolytic metabolism can directly influence intestinal inflammatory severity [32]. Nevertheless, several limitations should be acknowledged. Several limitations should be acknowledged. First, HIF-1α and glycolysis-associated proteins were measured in whole colorectal tissues rather than isolated macrophages, and pharmacological or genetic inhibition of HIF-1α or glycolytic enzymes was not performed. Therefore, macrophage-specific metabolic reprogramming and the causal role of the HIF-1α–glycolysis axis cannot yet be definitively established. Second, the present study did not directly assess glycolytic flux, mitochondrial respiration, or oxidative stress-related markers, limiting mechanistic interpretation of the observed metabolic alterations. Third, only male mice were included, which limits the generalizability of the findings to females. Future studies incorporating macrophage isolation or single-cell analysis, metabolic flux measurements, pathway-specific interventions, oxidative stress assessment, and both sexes will be needed to further clarify the mechanisms underlying chronic MC-LR-induced colorectal inflammation and metabolic remodeling.

## Figures and Tables

**Table 1:** Sequences of primers used for qPCR.

Gene	Forward Primer (5′→3′)	Reverse Primer (5′→3′)
*IL-1β*	AAGGGCTGCTTCCAAACTTTGAC	ATACTGCCTGCCTGAGCTCTTGT
*IL-6*	CCACGGCCTTCCCTACTTC	TTGGGAGTGGTATCCTCTGTGA
*TNF-α*	CCCACGTCGTAGCAAACCA	ACAAGGTACAACCCATCGGC
*β-Actin*	TCAAGATCATTGCTCCTCCTGAG	ACATCTGCTGGAAGGTGGACA

## Data Availability

The original contributions presented in this study are included in the article. Further inquiries can be directed to the corresponding author(s).
